# Floor Surfaces

**Published:** 1919-03-15

**Authors:** 


### HOSPITAL CONSTRUCTION: THE NEW ERA.

### IV. Floor Surfaces.

We are afraid it has to be admitted that the perfect material for a ward floor-surface still awaits discovery or invention. When we consider the several qualities that such a material has to combine, it is perhaps hardly to be wondered at that the faultless article is hard to find, or too costly to purchase. A ward floor should be (1) impervious, (2) not too hard. (3) elastic (4) warm, or at least not aggressively chilly, (5) not slippery, and (6) as a corollary to No. (1), as near as possible jointless. Probably rubber is the only material that comes within measurable distance of fulfilling all these conditions. Against its use is the consideration of cost, and perhaps that is really its only fault. We are familiar with its adaptation as a paving in banks where it is employed in the form of thin sheets. Its wearing power is great; if frequently washed it keeps clean and bright and, if its initial cost can be met, it will not call for the expense of frequent renewal. The ordinary domestic floor of deal boards on joists is not often found in hospitals for the reason that, in a modern institution, the structure of the floor is almost always of concrete. This construction is, of course, adopted for reasons of fire-prevention. Our attention is therefore confined to materials which can conveniently be laid upon a breeze or concrete floor. Plank floors, of deal or hard wood, are not excluded by these conditions, for there are three ways of attaching a plank floor to a concrete base. One, the simplest, is, if the concrete be composed of breeze, to nail the boards direct to the breeze itself. Another is to attach them to small battens embedded flush in the breeze, and the third consists of laying joists upon the surface of the breeze to which the boards are nailed as in the case of an ordinary joisted floor. Of course, a hollow space is left between each of the joists. The objection to the two first methods is that the boards, lying directly on the concrete, lose something of the resilience, which is one of the advantages of a plank floor, while the third method may fail owing to the fact that a hollow air-space enclosed by concrete beneath and wood above is liable to engender dry-rot—that most terrible foe of building owners. In any case, wherever boarded floors are used we are up against the danger that the joists may not, owing to the original sin, both of contractors and of timber, be perfectly tight: open joints, even if the boards are tongued, mean a lodgment for dirt and germs, and even washing only serves to entice the germs and the dirt into the crevices between the boards. Of block floors, *i.e.*, floors formed of pieces of timber measuring, say, 6″ by 3″ by 1½″ (deep), we shall speak later, merely remarking here that, if of deal or pitch pine, they should be specified to be "rift-sawn" and further, that a bad block-floor, in which the joints open through shrinkage or actually work loose, is the worst floor surface of all. The meaning of the "rift-sawn" specification is that the blocks should be so cut out of the solid baulk that the laminæ of the timber do not lie horizontal. If they do so lie, the upper laminæ, or flakes of the grain, are liable to be chipped off by ordinary foot traffic.

We next have to consider the various forms of mineral floors, which are chiefly mosaic or terrazzo, the latter being merely a cheaper form of mosaic, in which the pieces or tesseræ are not cut to regular squared sizes, but are laid at random, mixed in a sort of porridge of cement. This fills up the interstices of the blocks, which are, in effect, nothing more than waste marble chippings of irregular shapes and sizes. All these floors are excellent as fulfilling the conditions of impermeability, jointlessness, and cleanliness. They are perfect for corridors, waiting-halls, or operating-theatres, but they are complained of in wards as being too hard, too cold and too slippery for a patient who is not strong on his legs. Various experiments, some on a large scale, have been made with linoleum laid direct upon the smooth finished surface of the concrete floor. There is a great deal to be said for this material; for one thing, it is the joy of the char-women, for it is more easily washed than almost any other floor surface, and any failure to make it spotlessly clean is easily detected. It has joints, of course, but the joints are comparatively far apart and with careful laying and cutting can be reduced to a minimum. But linoleum, however thick, is not perfect. It does wear out, and, though not so chilly as the bare cement (or terrazzo) would be, it is not really warm. Again, though it tempers the hardness of the solid floor, it is not sufficiently elastic or resilient. Great hopes were raised some fifteen years ago or more by the introduction of a material variously named Papyrolith, Euboeolith, Stonwod or Armadek, which promised a smooth, non-metallic, jointless, and washable surface. It is compounded, we believe, of magnesia and sawdust, and can be tinted to any colour from bright red to a fierce arsenical green. As a surface it is certainly commendable, being reasonably solid and yet neither chilly nor uncompromisingly hard to the tread. But it has been found to possess deleterious qualities. Its chemical ingredients are sometimes fatal to any metal with which they may come into contact. It has been known in an operating theatre to consume waste and supply pipes which passed through it, and it has even corroded the structural metal of the floor itself. We have also known it to fight a successful battle with the plaster on the walls in a case where it was carried up the lower part of a corridor wall to form a coved skirting.

What, then, among all these materials against which complaints can be honestly laid, are we to choose as the best if not the ideal floor? In spite of all that has been said we believe that a certain class of wood-block floor best fulfils general require-

☛ The previous articles appeared on January 25, February 8, and March 1, p. 480.

**Hospital Construction**—(*continued*).

ments. We allude to the use of good hardwood blocks laid diagonally—in "herring bone" pattern—and continually polished with a good beeswax polish such as "Ronuk." Hardwood, such as oak, provides blocks which if properly seasoned can be relied upon not to shrink unduly, and the polish if properly and frequently applied overcomes one of the two objections that can be argued against the material. Being laid direct upon the concrete, save for a bed of bituminous mastic, the surface is still only moderately yielding to the tread. But the worst fault of a block floor—the many joints—is practically combatted by the fact that the polish works into the interstices, and so prevents the formation of harbourage for microbes. Moreover, the polish has a moderately antiseptic effect, and the housekeeping staff take a certain pleasure in the polishing process which is less onerous than the ordinary floor scrubbing. Certainly the application of the polish is a preservative to the wood, and the same can by no means be said of the ordinary soap-and-water process.

Probably, if perfect comfort be aimed at, the best attainable floor short of the above-mentioned rubber sheeting would be made by using narrow planks of teak carefully tongued and closely wedged up. These would be nailed to battens placed about two feet apart in place of the normal 12 inches, thus obtaining the spring of a ball-room floor. The wax polish treatment would then be applied and the only possible objection would be the unventilated air space under the floor. The dry rot danger might be forestalled by washing the battens with corrosive sublimate and carefully removing all shavings and wood chippings.

